# Supercapacitor Model with Charge-Dependent Parameters Based on Experimental Frequency Response

**DOI:** 10.3390/s26072075

**Published:** 2026-03-26

**Authors:** Carlos Gilabert-Torres, Sergio Ignacio Serna-Garcés, Carlos Andrés Ramos-Paja, Juan Domingo Aguilar-Peña, Catalina Rus-Casas

**Affiliations:** 1 Electronic Engineering and Automatic Department, University of Jaen, Las Lagunillas Campus, A3 Building, 23071 Jaen, Spain; gilabert@ujaen.es (C.G.-T.); jaguilar@ujaen.es (J.D.A.-P.); crus@ujaen.es (C.R.-C.); 2 Centre for Advanced Studies in Energy and Environment CEACTEMA, Universidad de Jaén, 23071 Jaen, Spain; 3 Departamento de Electrónica y Telecomunicaciones, Institución Universitaria ITM, Medellín 050013, Colombia; 4 Facultad de Minas, Universidad Nacional de Colombia, Medellín 050041, Colombia; caramosp@unal.edu.co

**Keywords:** supercapacitor, supercapacitor equivalent circuit, frequency response analysis, modeling, energy storage, electrochemical impedance spectroscopy, electric double-layer capacitors

## Abstract

The characterization of supercapacitors (SCs), particularly electric double-layer capacitors (EDLCs), is useful for the design of energy management systems. This article presents a five-parameter dynamic model based on electrochemical impedance spectroscopy (EIS) data. Unlike conventional fixed-parameter models, this study explores an approach that accounts for voltage dependence. The model was evaluated using four commercial 58-farad SCs over a frequency range of 10 mHz to 300 kHz and at voltages ranging from 6.25% to 93.75% of the nominal value. The results show that the parameters vary with the state of charge; for example, the effective capacitance increased by up to 24% when moving from 6.25% to 50% of the nominal voltage. The model, fitted using nonlinear optimization algorithms, has a mean square percentage error (MSPE) of less than 3%. This approach estimates the dynamic behavior of the SCs, facilitating the simulation and tuning of management and protection strategies.

## 1. Introduction

Global decarbonization has drastically accelerated the adoption of renewable energy. Annual renewable capacity addition rose from 290 GW in 2020 to 750 GW in 2025, with a projected addition of 4600 GW between 2025 and 2030 [1]. However, the intermittent nature of these sources challenges grid stability and reliability [2,3]. Energy storage systems (ESSs) are essential for mitigating this instability and managing the injection of renewable energy into the grid [4,5,6]. The main ESS technologies include thermal, mechanical, and electrochemical storage [7,8]. The latter primarily comprises batteries and supercapacitors (SCs), whose technical characteristics are compared in Table 1.

Batteries are distinguished by their high energy density, whereas SCs excel in power density. This distinction makes batteries optimal for bulk energy storage. In contrast, SCs (featuring 85–98% efficiency and a lifespan of up to one million cycles) are well-suited for frequency and voltage regulation applications. Therefore, the complementarity nature of those systems is essential for the decarbonization of the power supply [9,10]. SCs are categorized into electric double-layer capacitors (EDLCs), pseudocapacitors, and hybrid capacitors [11,12]. EDLCs rely on electrostatic double-layer storage using porous carbons. They are characterized by a long life cycle, high power, and stability, albeit with limited specific energy [13]. In contrast, pseudocapacitors incorporate faradaic materials, such as metal oxides or conducting polymers, to achieve higher capacitance and energy. However, this comes at the cost of lower power and reduced life cycle due to structural degradation from surface reactions [14]. Hybrid SCs combine an EDLC electrode with a pseudo-capacitive one to balance energy and power, offering higher voltages, yet their complexity and durability depend heavily on the specific chemistry [15]. Currently, EDLCs dominate power systems due to their technological maturity and superior stability [16,17].

**Table 1:** Characteristics of the main electrochemical energy storage systems.

ESS Type	Technology	Energy Density (Wh/kg)	Power Density (W/kg)	Lifespan (10^3^ Cycles)	Ref.
SCs	EDLC	5–10	5000–15,000	100–1000	[18,19,20]
	Hybrid	10–100	1000–3000	10–100	
	Pseudocapacitor	10–30	2000–6000	1–10	
Li-ion batteries	LFP	90–250	1000–2000	3–5	[19,21,22]
	NMC	150–260	1000–2000	1–2	
	LTO	60–150	2000–3000	7–10	

Effective integration of SCs into power systems, particularly for real-time control, demands accurate models. Model development proceeds from experimental testing under various conditions to propose an equivalent circuit, followed by parameterization and validation. This characterization relies on three standard techniques: cyclic voltammetry (CV) evaluates capacitance and charge/discharge behavior [23,24]; galvanostatic charge–discharge (GCD) determines internal resistance and energy efficiency [25]; and electrochemical impedance spectroscopy (EIS) analyzes frequency-dependent diffusion and capacitance to identify critical model parameters [26,27].

**Figure 1:**
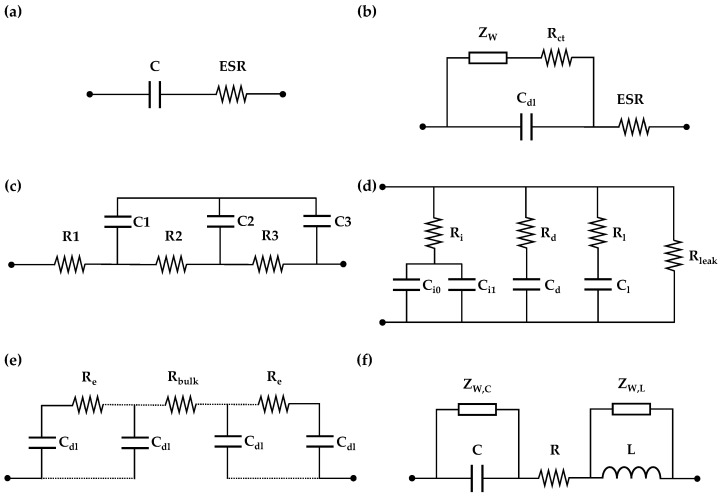
Supercapacitor models: (**a**) RC; (**b**) Randles; (**c**) Ladder; (**d**) Zubieta; (**e**) TLM; (**f**) WE-RLC.

Simplified SC models, such as the RC circuit, are suitable for power management studies characterized by slow dynamics [28]. Conversely, switching converter design and real-time control strategies require detailed representations to capture fast transients across a broad frequency spectrum [29]. Inaccurate modeling can lead to improper device sizing (increasing costs or compromising safety) and result in unstable controllers [30]. Figure 1 summarizes the main SC models reported in the literature.

The Randles model (Figure 1b) incorporates the Warburg element Z_W_ to model ionic diffusion within the porous structure and the electrolyte. Although this approach offers higher accuracy than the RC model (Figure 1a) and is widely used in EIS [31,32], it has limitations like ignoring the effect of applied voltage on the capacitance and ESR or not accounting for inductive effects from cable connection or non-ideal behavior in the electrolyte. The Ladder (Figure 1c) and Transmission Line (TLM, Figure 1e) models accurately describe electrode porosity; however, their high computational cost makes them impractical for real-time simulations [33]. Similarly, the Zubieta model (Figure 1d) improves the long-term charge redistribution response but is limited at high frequencies [33]. Notably, none of these models capture the inductive behavior observed in SCs at high frequencies [29,30]. In contrast, the Warburg-Enhanced RLC model (WE-RLC, Figure 1f) [30] presents three distinct advantages over traditional approaches: it incorporates high-frequency inductive behavior, enhances representation accuracy via Warburg elements, and facilitates parameterization and implementation in simulation software (MATLAB-2025a).

Accurate parameter determination in SC models (such as capacitance and equivalent series resistance (ESR)) is critical for predicting device dynamic behavior. Currently, analytical methods and least squares optimization (LSO) represent the standard approach for parameter extraction using time domain tests [34,35]. Conversely, applications requiring real-time monitoring of degradation or state of charge employ recursive least squares (RLS) and Subspace System Identification (SSID) techniques. Those methods allow for automatic model state matrix updates without manual tuning [36,37]. Furthermore, the increasing complexity of fractional-order and multi-pore models has driven the adoption of metaheuristic optimization, including particle swarm optimization (PSO) and genetic algorithms (GAs), which demonstrate superior robustness against system nonlinearities [38]. Table 2 compares current modeling strategies and their main characteristics.

Most existing literature models EDLCs using experimental data from CV and GCD. However, modeling for switching systems requires frequency analysis, which relies on EIS. Previous studies [29,39,40] show that EDLC behavior varies with temperature and charge, proposing variable-parameter models to improve accuracy across the operating range. Nevertheless, those approaches are largely limited to the time domain or require specialized software to parameterize complex models [40]. To address the lack of simple, frequency-response-based models, this paper analyzes impedance data from four identical EDLCs using a frequency response analyzer (FRA) ranging from 0.01 Hz to 300 kHz. Moreover, it proposes a dynamic model with five parameters that depend on the state of charge (SOC), which is represented by the bias voltage. This approach facilitates parameterization and ensures accuracy across wide frequency and load ranges, which enable its application in renewable energy systems.

The rest of the paper is organized as follows: Section 2 describes the materials and methodology, including the EIS test setup, the parasitic component correction procedure, and the model parameterization workflow; Section 3 presents the frequency response results at different voltage levels and validates the accuracy of the dynamic model; Section 4 discusses the robustness of the experimental and modeling methods, comparing the results with the existing literature; and finally, Section 5 summarizes the main conclusions and highlights their relevance for optimizing energy management systems with high renewable penetration.

**Table 2:** Literature review.

Ref.	SC Technol.	Exptl. Method	Analysis Domain	SC Model	Parameter-Varying Model	Parameterization Method
[41]	EDLC	CV + GCD	Time	Simplified Randles	No	GRG
[42]	Hybrid	CV	Time	RC + diffusion element	Yes	GRG
[43]	Pseudocapacitor	CV + GCD	Time	ML	N/A	RF + CART
[39]	EDLC	GCD	Time	Fractional-order Randles + leakage resistance	Yes	PSO
[35]	EDLC	GCD + self-discharge	Time	Zubieta–Bonert + Voltage-dependent resistor and capacitor	Yes	NLO + CTRR
[44]	EDLC	GCD	Time	Fifth-order equivalent circuit	Yes	Stepwise + NLO
[29]	Hybrid	EIS	Frequency	Integer-order model (Voigt) + fractional-order model	No	GA + NLO
[45]	EDLC	GCD	Time	Dynamic fractional-order model	Yes	LSO
[40]	EDLC	EIS	Frequency	Eleven-parameter equivalent electrical circuit	Yes	Specialized software + statistical analysis
[46]	EDLC	GCD	Time	Two-branch Zubieta model	Yes	Direct analysis of experimental data
[27]	EDLC	CV + EIS	Time + frequency	Physics-based model	No	Iterative manual parameterization
[30]	EDLC	EIS	Frequency	WE-RLC	No	Direct analysis of experimental data
This work	EDLC	EIS	Frequency	Charge-dependent WE-RLC	Yes	Stepwise + NLO

## 2. Materials and Methods

Electrochemical impedance spectroscopy is a key non-invasive technique for characterizing SCs. This method applies a small alternating current (AC) or voltage perturbation across a frequency range to analyze the phase and amplitude response. This determines the impedance transfer function of the system, which is essential for modeling SC behavior.

### 2.1. Materials

**Figure 2:**
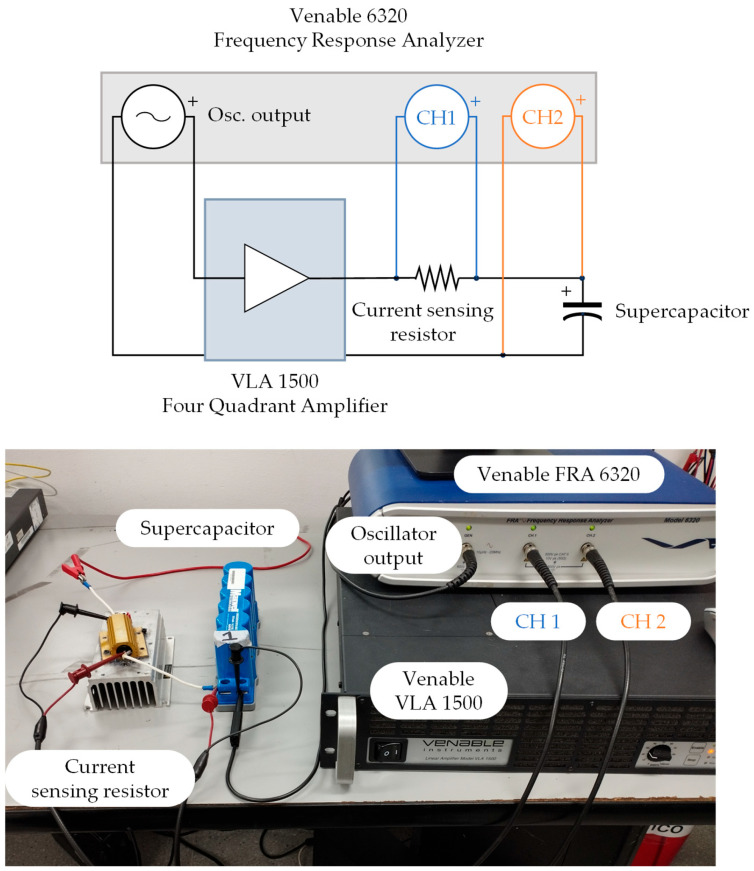
Schematic and experimental setup.

To obtain robust EIS data and develop the variable-parameter SC model, four units of the commercial EDLC BMOD0058-E016-B02 (Maxwell Technologies, Glendale, CA, USA) [47] were tested. This device was selected due to its widespread use in power electronics and energy management applications, such as DC bus stabilization in microgrids and frequency regulation [48,49,50]. The EIS measurements were performed using a Venable 6320 FRA (Venable Instruments, Austin, TX, USA) [51] coupled with a Venable VLA 1500 four-quadrant amplifier (Venable Instruments, Austin, TX, USA) [52]. Figure 2 illustrates the schematic and experimental setup, and the specifications of the equipment used are shown in Table 3.

The FRA 6320 and VLA 1500 were combined to test the device across its full operating voltage range (0–16 V), as the VLA amplifies the bias voltage beyond the limit of the FRA (10 V). Additionally, the Venable 6320 FRA features a servo function that dynamically adjusts the output amplitude of the voltage perturbation signal (Osc. Output) to achieve the target amplitude at the SC (FRA Channel 2). Integrating the VLA 1500 amplifier allows for increasing the amplitude of the perturbation to compensate for the voltage divider formed by the SC and the current-sensing resistor. The combination of the FRA 6320 servo function and the VLA 1500 gain enabled the disturbance in the SC to be within the range of 1–10 mVrms during the tests. Consequently, the experimental setup was configured in accordance with the guidelines outlined in the extant literature, as detailed in the subsequent section.

#### 2.1.1. Optimal Test Conditions

The international standard UNE-EN IEC 62391 defines testing and classification procedures for EDLCs [53,54]. Although it does not specify conditions for performing EIS, Annex B of UNE-EN IEC 62391-1 [53] provides guidelines for AC testing. Table 4 summarizes the recommendations found in the literature, where $U_{N}$ is the rated voltage of the SC.

A wide frequency range allows for complete system characterization: low frequencies reveal slow processes (diffusion and charge accumulation), while medium-to-high frequencies exhibit rapid processes (ohmic resistance and inductive behavior) [56]. The AC perturbation amplitude must be a trade-off: low enough to ensure impedance linearity, yet high enough to maintain an acceptable signal-to-noise ratio (SNR) [57,58]. Integration time is a critical parameter for optimizing SNR; increasing the number of measurement cycles enhances signal quality at the expense of longer experiment duration. To verify the linearity and stability of the SC response during EIS, multiple tests were conducted using various perturbation amplitudes and integration times (Appendix A reports those additional experiments).

Low-frequency testing requires significantly longer durations, particularly when multiple measurement cycles are involved. This prolonged duration, combined with potential calibration errors, may induce DC voltage drift in the supercapacitor. Consequently, selecting an integration time that balances an adequate SNR with minimal test duration is critical. To enhance flexibility in parameter optimization, the frequency spectrum was divided into two ranges: low (10 mHz–1 Hz) and medium–high (1 Hz–300 kHz). Another key temporal parameter is the AC stabilization time (delay), which mitigates measurement transients caused by frequency shifts. Regarding experimental conditions, the ambient temperature must remain stable near 20 °C to minimize thermal variations in the SC parameters [54].

#### 2.1.2. Characterization of Parasitic Circuit Components

**Figure 3:**
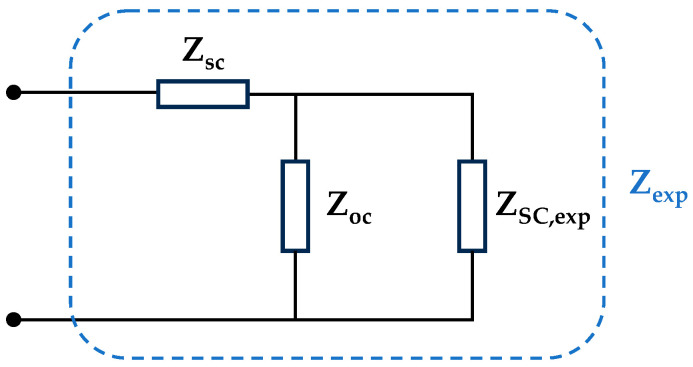
Parasitic components of the test circuit.

The test circuit in Figure 2 yields the experimental impedance, $Z_{exp}$. However, the setup introduces parasitic components that affect measurements across frequencies. As depicted in Figure 3, these components are modeled as series $Z_{sc}$ and parallel $Z_{oc}$ impedances relative to the supercapacitor ($Z_{SC,exp}$).

To isolate the supercapacitor impedance, the parasitic circuit components must be quantified and compensated. Thus, short-circuit and open-circuit measurements are performed, under the conditions listed in Table 4, to obtain $Z_{sc}$ and $Z_{oc}$, respectively. Finally, the corrected impedance is calculated using Equation (1).

(1) $$Z_{SC,exp}=\frac{\left(Z_{exp}-Z_{sc}\right)\cdot Z_{oc}}{Z_{sc}+Z_{oc}-Z_{exp}}$$

The elimination of those parasitic components ensures more accurate and easier-to-reproduce results when using alternative test circuits.

### 2.2. Experimental Methodology

**Figure 4:**
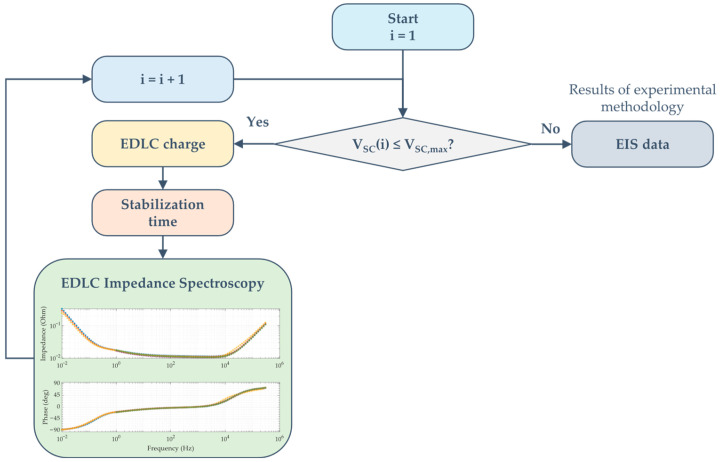
Experimental methodology workflow.

Figure 4 presents a flowchart illustrating the testing methodology employed in this study.

First, the uncharged supercapacitor was charged to the target voltage using a current-limited external DC source to reduce charging time. Next, the SC was connected as shown in Figure 2. A bias voltage was set on the Venable 6320 to match the target terminal voltage. The device was maintained under these conditions for 1 h to ensure a steady state, achieving a bias voltage stability of ±1%. Finally, EIS was performed by sweeping the perturbation frequency while recording voltages on channels 1 and 2 of the FRA. The experimental impedance $Z_{exp}$ was calculated for each frequency point ∀k=1, 2,…,N according to Equation (2), where $V_{CH1}$ and $V_{CH2}$ denote the effective voltages measured in channels 1 and 2, respectively, and $R_{s}$ represents the current-sensing resistor.

(2) $$Z_{exp,k}=\frac{V_{CH2,k}}{V_{CH1,k}}\cdot R_{s}$$

After obtaining the impedance curve for a specific load voltage, the procedure was repeated across multiple DC voltage levels to fully characterize the SC range.

### 2.3. Modeling Methodology

**Figure 5:**
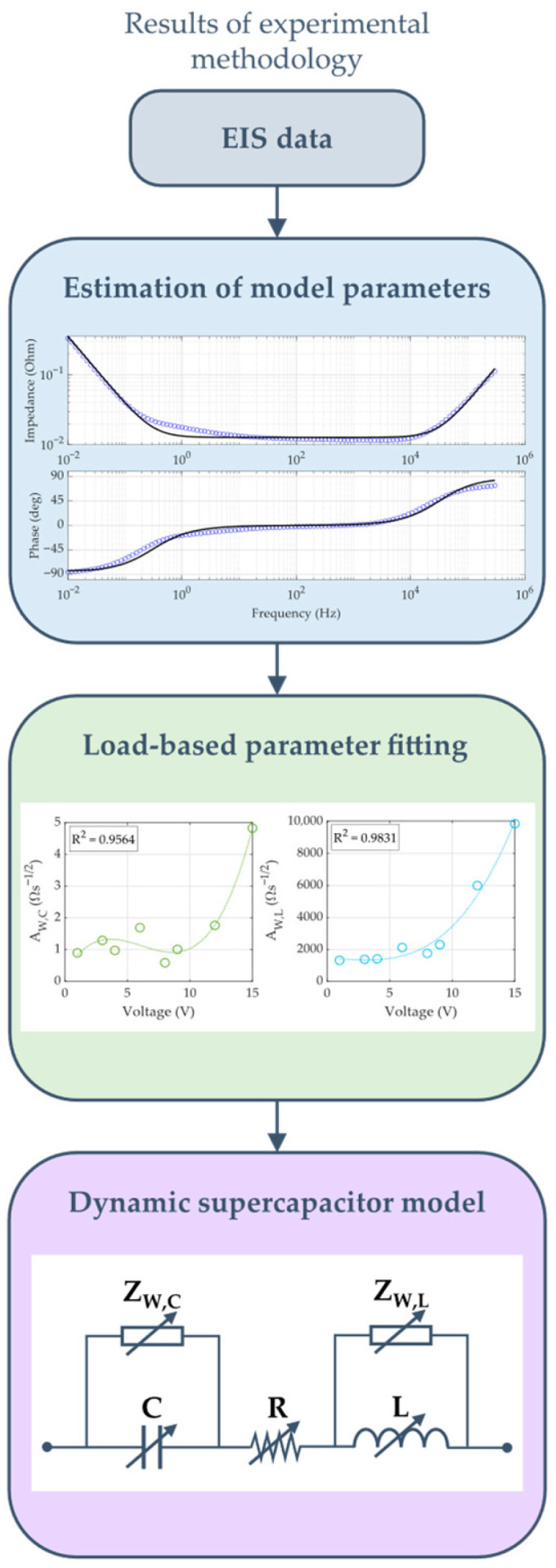
Modeling methodology workflow.

The methodology employed in modeling the frequency response of the supercapacitor is illustrated in the flowchart presented in Figure 5.

First, the SC model parameters are estimated for each SOC using experimental impedance curves. Subsequently, fitting equations are calculated, which express the model parameters as a function of the SC bias voltage. The resulting model determines the impedance based on the frequency and charge level of the EDLC.

#### 2.3.1. Dynamic Equivalent Circuit Model of a Supercapacitor

**Figure 6:**
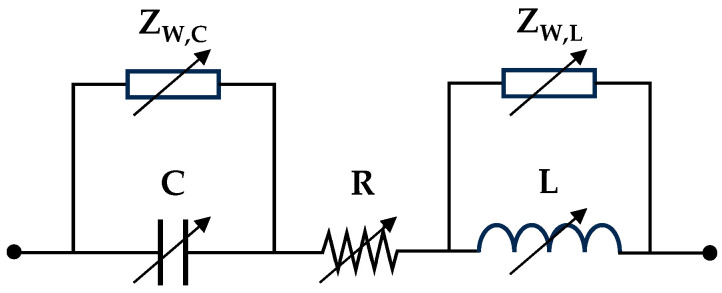
Supercapacitor circuital equivalent.

The literature offers multiple equivalent circuits for SCs. The five-parameter model described in [30], combining RLC and Warburg elements, accurately models SCs across a wide frequency range. Therefore, the WE-RLC structure was selected as the basis for a variable-parameter model capable of reproducing the SC response across its entire charge spectrum. Figure 6 shows the proposed model.

The WE-RLC model defines five parameters linked to the internal dynamics of the supercapacitor. Resistance (R) represents the equivalent series resistance, which encompasses the ohmic losses of the conductors. Capacitance (C) models charge storage in the electric double layer. To capture high-frequency behavior (typically >10 kHz), the model incorporates an inductance (L) that reflects the parasitic effects of the terminals and the internal geometry of the device. Additionally, two Warburg impedances are incorporated in parallel with C and L, thereby representing the internal diffusion phenomena. Equation (3) defines the semi-infinite Warburg impedance ($Z_{W}$) using the Warburg coefficient ($A_{W}$), where ω is the angular frequency of the electrical system [60,61].

(3) $$Z_{W}=A_{W}/\sqrt{j\omega }$$

To model the impedance phase adjustment, the equivalent circuit includes two Warburg elements: one in parallel with the capacitor ($Z_{W,C})$ and another with the inductor ($Z_{W,L})$. The implementation of these elements in simulation software (MATLAB-2025a) is described in [30]. However, while the previous WE-RLC model [30] was derived at only 50% of the rated voltage, this study analyzes the SC at multiple charge levels using EIS. Therefore, the device parameters (R, L, C, $A_{W,C}$ y $A_{W,L}$) are estimated across multiple operating points and fitted to an n-degree polynomial function of the SC voltage ($V_{SC}$), as shown in Equation (4), where P is the model parameter and $a_{i=0\ldots n}$ are the coefficients of the equation.

(4) $$P\left(V_{SC}\right)=a_{0}+a_{1}\cdot V_{SC}+a_{2}\cdot V_{SC}^{2}+\ldots +a_{n}\cdot V_{SC}^{n}$$

The polynomial degree is increased, starting from zero, until reaching a coefficient of determination higher than 0.9. The coefficient of determination, denoted as $R^{2}$, is a statistical measure that indicates the proportion of the total variance of the dependent variable that is explained by the proposed model, and it ranges from 0 to 1. Thus, this threshold ensures an accurate fit while maintaining model simplicity. Since the parameters are positive, the equations must be constrained to non-negative values within the SC voltage range. Equation (5) yields the impedance for the model shown in Figure 6.

(5) $$Z_{m}(\omega ,V_{SC})=R+\frac{Z_{W,C}(V_{SC})}{Z_{W,C}(V_{SC})\cdot j\omega C(V_{SC})+1}+\frac{Z_{W,L}(V_{SC})\cdot j\omega L(V_{SC})}{Z_{W,L}(V_{SC})+j\omega L(V_{SC})}$$

Subsequently, the value of the model parameters is optimized to adjust its response to the experimental data.

#### 2.3.2. Parameterization of the Model

**Figure 7:**
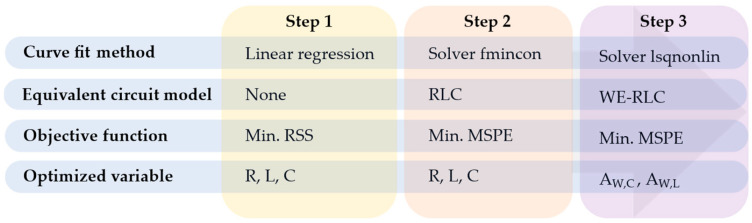
Equivalent circuit parametrization process.

The parameterization starts obtaining N impedance points across a frequency range for each SC SOC. Subsequently, the WE-RLC model parameters (R, L, C, $A_{W,C}$ y $A_{W,L}$) are estimated using a stepwise optimization algorithm, as illustrated in Figure 7.

In Step 1, the initial R-L-C parameters were determined via linear regression, minimizing the residual sum of squares (RSS) regarding the experimental impedance magnitude of the EDLC. This regression splits the frequency range into three segments with distinct behaviors, as shown in Table 5.

In Step 2, the RLC model was fitted to the experimental impedance data ($Z_{SC,exp}$) using the MATLAB-2025a fmincon solver. This procedure estimates R, L and C by minimizing the Mean Squared Percentage Error (MSPE), as defined in Equation (6).

(6) $$MSPE=\frac{100}{N}\sum_{k=1}^{N}{\left(\frac{\left|Z_{SC,exp,k}-Z_{SC,model,k}\right|}{\left|Z_{SC,exp,k}\right|}\right)}^{2}$$

The R-L-C values obtained in Step 1 are used as initial parameters, and we set the limits listed in Table 6.

The maximum ESR (R) and capacitance reported for the tested EDLC are 22 mΩ and 70 F, respectively [47]. The lower capacitance limit is calculated, considering that values at nominal voltage are up to 60% higher than those at low charge [62]. Since resistance at medium–high frequencies decreases significantly compared to low frequencies [40], the lower limit of R is set at 6 mΩ. The inductance limits were obtained iteratively from experimental data. This second step yields R, L and C parameters that fit a simple equivalent circuit model, which serves as an ideal initialization point for more complex models. Finally, the experimental impedance is fitted to the WE-RLC model. In Step 3, the parameters $A_{w,C}$ and $A_{w,L}$ are optimized using the MATLAB lsqnonlin solver. This function employs the Interior-Point algorithm [63] with a maximum of 2000 iterations. Table 7 shows the solver configuration used in Step 3.

The literature values for $A_{W,C}$ typically range from 0.1 to 7 Ωs^−1/2^, reaching up to 20 Ωs^−1/2^ in supercapacitors with extremely small pores (<1 nm) [64,65]. For $A_{W,L}$, an iterative process was used to determine the initial values and limits from experimental data. Since the solution depends on the initial estimation, previous studies employed heuristics such as genetic algorithms [29]. Here, an analytical method was proposed: Steps 1 and 2 are used to approximate an optimal starting point. Subsequently, in Step 3, a sweep of R-L-C combinations derived from Step 2 was performed (using a 5% tolerance and a 1% step size). This strategy enhances the method’s robustness against local minima [44].

To validate the variable parameter model, its accuracy was assessed against experimental data. Since impedance is a complex variable, the SC model error is decomposed into two components: distance error ($e_{d}$) and angle error ($e_{\theta }$). The former quantifies the magnitude difference between theoretical and experimental magnitudes, whereas the latter represents the phase difference, as shown in Equations (7) and (8).

(7) $$e_{d}=\frac{\sum_{k=1}^{N}\left|Z_{SC,model,k}-Z_{SC,exp,k}\right|}{N}$$

(8) $$e_{\theta }=\frac{\sum_{k=1}^{N}arg(Z_{SC,model,k}-Z_{SC,exp,k})}{N}$$

The accuracy of the SC model is optimized when errors in both absolute ($e_{d}$ and $e_{\theta }$) and relative terms (MPSE) are minimal.

## 3. Results

This section presents the experimental impedance results and analyzes the SC curves at different charge levels. Subsequently, the WE-RLC model is fitted to represent the experimental data, and the errors are quantified. Finally, the parameter equations, as a function of bias voltage, are calculated and the accuracy of the proposed model is evaluated.

### 3.1. Characterization of the Parasitic Components in the Experimental Setup

**Figure 8:**
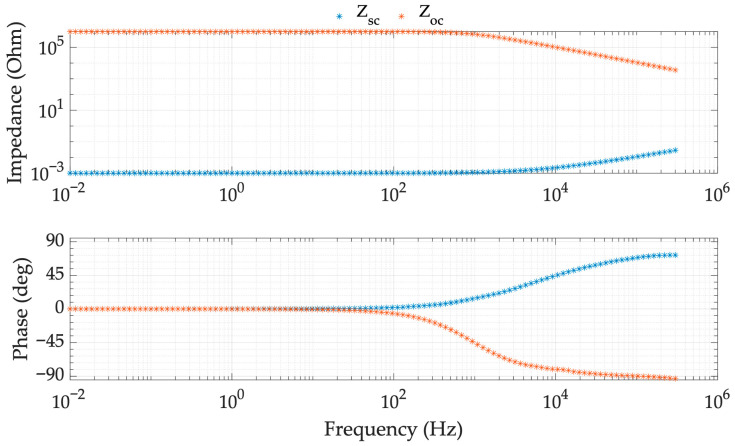
Parasitic impedances of the measurement circuit.

The measurement circuit was characterized by two tests without the SC: open-circuit and short-circuit. These tests determined the series and parallel parasitic impedances, which are shown in Figure 8.

The parallel impedance $Z_{oc}$ of the measurement circuit is close to 1 MΩ at frequencies below 10 kHz, dropping to a minimum of 3.6 kΩ at 300 kHz. Although the system acts as an open circuit below 10 kHz, impedance decreases sharply at higher frequencies, causing significant leakage current ($Z_{oc}$ in Figure 3). Conversely, the series impedance $Z_{sc}$ is resistive below 10 kHz with a value of approximately 1 mΩ, which is equivalent to approximately 9% of the actual impedance of the EDLC within that specified frequency range. At higher frequencies, the circuit becomes inductive, reaching a maximum of 29 mΩ at 300 kHz, which accounts for 23% of the mean EDLC impedance under load. Consequently, compensating for these parasitic components is critical in obtaining a truly representative and replicable device impedance.

### 3.2. EIS of a Supercapacitor at Different Charge Levels

**Figure 9:**
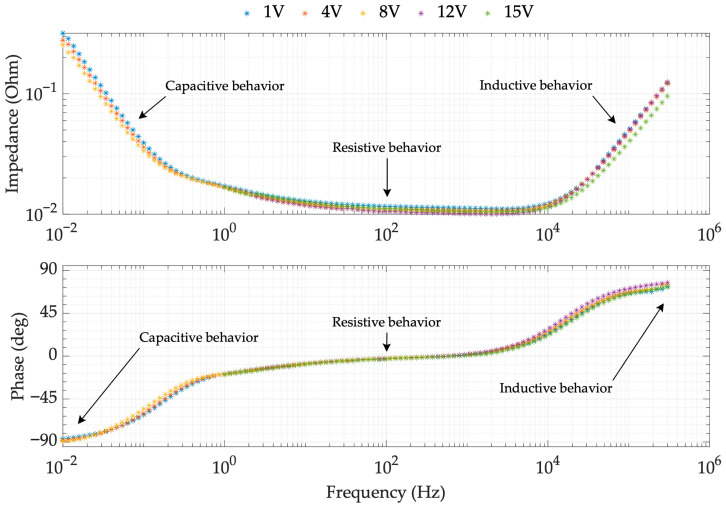
Frequency response of SC 1 at different load levels.

To obtain the EIS spectrum for the SC BMOD0058-E016-B02, the experimental setup detailed in Figure 2 and the parameters listed in Table 4 were adopted. Four identical EDLC units were tested to ensure statistical robustness. The reported impedance values were corrected by compensating for the parasitic impedances in the measurement circuit (Equation (1)), using the $Z_{oc}$ and $Z_{sc}$ reference measurements described previously. Tests covered bias voltages of 1 V, 4 V, 8 V, 12 V and 15 V (6.25–93.75% of the rated voltage). The frequency sweep ranged from 10 mHz to 300 kHz; however, for 12 and 15 V, the lower limit was restricted to 1 Hz due to limitations of the Venable 6320 FRA equipment. Figure 9 shows the frequency response for the first supercapacitor (SC 1) under those SOCs.

The impedance data reveal three distinct frequency behaviors: capacitive (<1 Hz), predominantly resistive (1 Hz–10 kHz), and inductive (>10 kHz). These findings justify including R, L, and C elements in the SC equivalent circuit to accurately represent its response.

Regarding the charge level, it is observed that capacitance increases at low frequencies as the charge increases. This behavior is consistent across the other three devices studied SC 2, SC 3 and SC 4 (those frequency responses are reported in Appendix B). In contrast, resistance and inductance showed minor variations with no clear trend. Unlike literature models that assume constant parameters, these results demonstrate a consistent and significant dependency on the charge level, particularly for the capacitance. The following section details the model parameterization and the obtained values.

### 3.3. Optimization of WE-RLC Model Parameters

**Figure 10:**
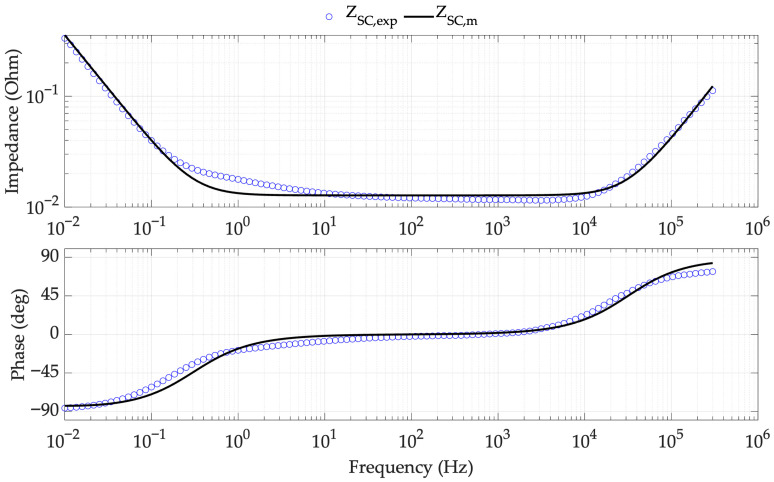
WE-RLC model fitting to experimental data from SC 1 with a bias voltage of 1 V.

**Figure 11:**
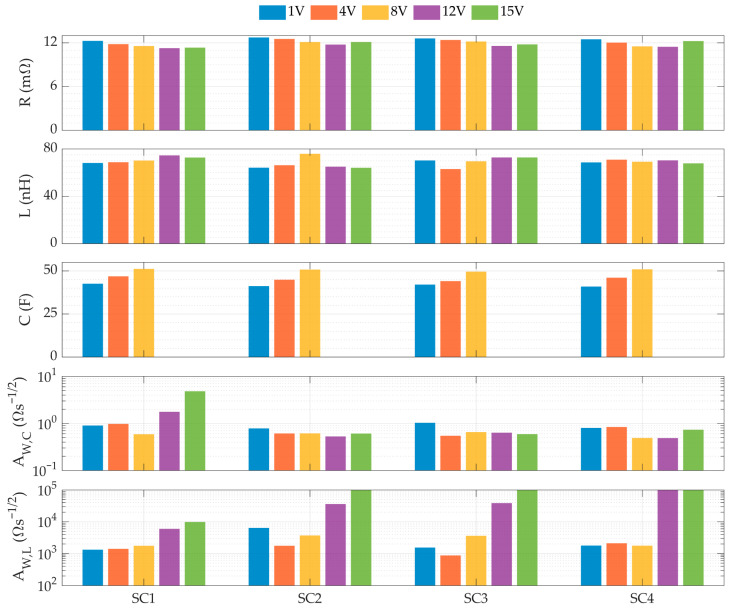
WE-RLC model parameters for different load levels of SCs 1–4.

The SC model parameters are obtained by fitting the experimental data according to the methodology proposed in Figure 7. Figure 10 illustrates this fit for SC 1 at 1 V, while Figure 11 summarizes the resulting parameters at different voltage levels for the various SCs.

Capacitance increased by 18–24% as the EDLC bias voltage rose from 1 V to 8 V. This increase followed a linear trend across all four devices, consistent with previous studies [42]. Although the minimum frequency limit (1 Hz) prevented capacitance calculation at 12 V and 15 V, results are consistent with the manufacturer’s rated value (58 F), determined at 50–100% of the rated voltage [47]. Resistance followed a parabolic trend, reaching a minimum at 12 V (a 7.6–8.2% decrease relative to 1 V). Inductance was in the range of 63–76 nH across voltage levels, with a mean of 69 nH and a standard deviation of 3.5 nH for the four EDLCs. Warburg coefficients $A_{W,C}$ and $A_{W,L}$ ranged from 0.5 to 5 Ωs^−1/2^ and 10^3^ and 10^5^ Ωs^−1/2^, respectively. While $A_{W,C}$ values remained stable in all four SCs across voltage levels, $A_{W,L}$ was significantly higher at 12 and 15 V. This indicates that the Warburg element $Z_{W,L}$, used to fit the high-frequency phase, becomes dominant at higher voltages. This is attributed to high-voltage side effects, such as electrolyte decomposition, carbon oxidation, and pore blocking [66].

The maximum mean errors for magnitude (5.03 mΩ) and phase (5.3°) remained low and stable across different SC charge levels. The MSPE peaked at 1.77%, confirming that the optimization algorithm minimized errors in both absolute and relative terms. Consequently, the WE-RLC model accurately fits the experimental data across all states of charge. Finally, the variable parameter model, based on the voltage dependence of R, L, C, $A_{W,C}$ and $A_{W,L}$, is derived in the following subsection.

Table 8 shows the distance and angle errors of the WE-RLC model alongside the MSPE.

### 3.4. Dynamic Supercapacitor Model

**Figure 12:**
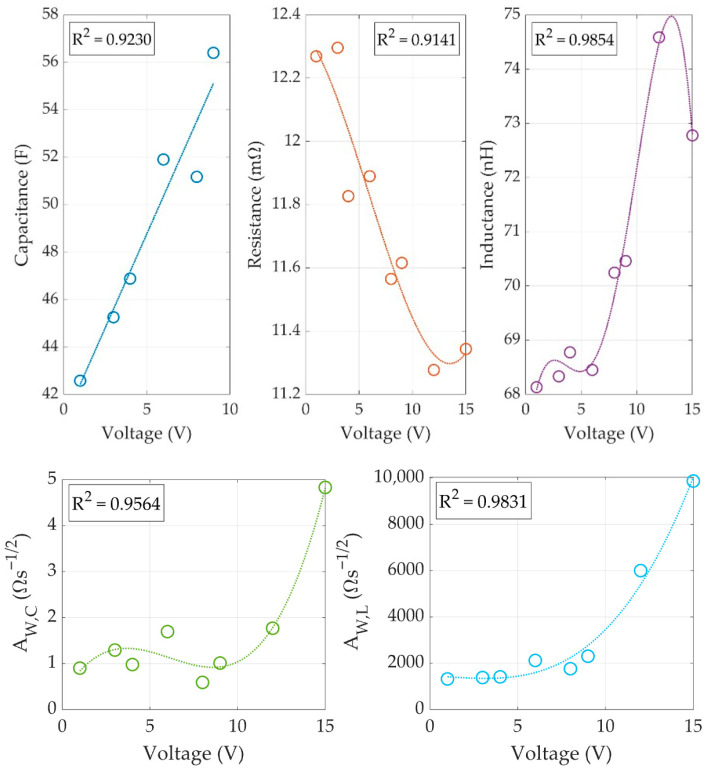
Polynomial fitting of WE-RLC model parameters as a function of the SC bias voltage.

The proposed dynamic SC model comprises five voltage-dependent parameters (R, L, C, $A_{W,C}$ y $A_{W,L}$). Based on the previous data, a polynomial fitting is performed for those parameters regarding the SC voltage, following the method introduced in Section 2.3. To illustrate this process using SC 1, the device was tested at additional charging voltages of 3 V, 6 V, and 9 V to increase data resolution. Figure 12 displays the resulting fitting curves.

Polynomial fits applied were first-order for capacitance, third-order for resistance and Warburg components, and fourth-order for inductance. The obtained R^2^ values (0.9141–0.9854) indicate a reliable fit to the experimental data. Table 9 lists the coefficients of the fitting equations, generalized in Equation (4), for each parameter.

**Figure 13:**
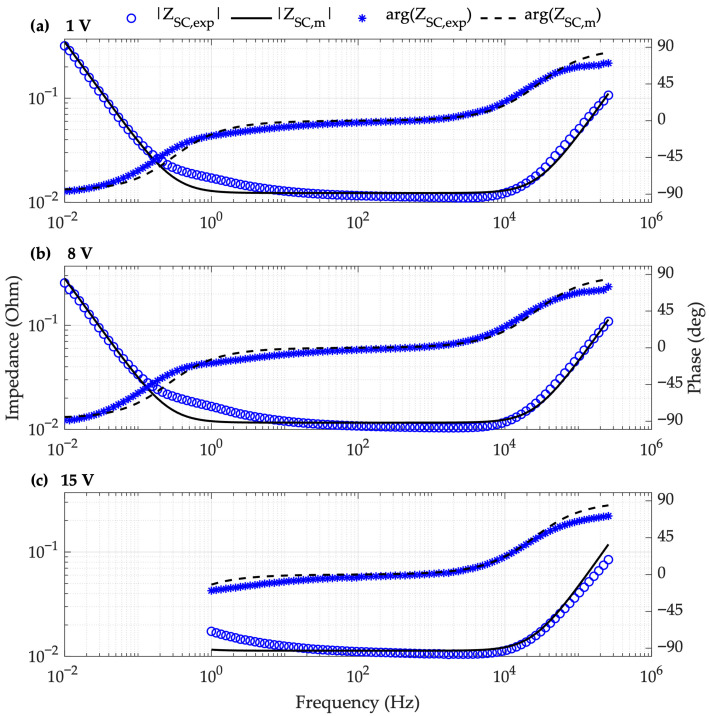
Experimental and theoretical frequency response of SC 1 for load voltages of (**a**) 1 V, (**b**) 8 V, and (**c**) 15 V.

These equations determine the model parameters as a function of the SC voltage. To validate the model, we compared the calculated impedance with experimental data at various SOCs. Figure 13 shows the experimental ($Z_{SC,exp}$) and modeled ($Z_{SC,m}$) frequency responses of SC 1 at 6.25% (1 V), 50% (8 V), and 93.75% (15 V) of $U_{N}$ (16 V).

The variable parameter model reproduces the experimental impedance across most of the frequency range. The largest discrepancies occur at medium–low (0.05–5 Hz) and high frequencies (>100 kHz). Table 10 summarizes the $e_{d}$, $e_{\theta }$, and MSPE values for different states of charge.

Results indicate that at high SOC levels, the distance error decreases to 2.805 mΩ while the angle error increases to 5.752°. The MSPE ranges between 2.28% and 3.03%, with a variation coefficient equal to 13%, resulting in low and similar values for different bias voltages. These findings demonstrate that the model accurately represents the SC behavior throughout its entire operating range. Consequently, the proposed model is suitable for dynamic response studies and control system applications requiring high fidelity across a broad operating and frequency range.

## 4. Discussion

### 4.1. EIS of the Supercapacitor

EIS characterization was performed for the EDLCs following the recommendations summarized in Table 4. The adopted Venable 6320 FRA was previously validated for SCs characterization [30]. The extant literature recommends the use of a potentiostat in conjunction with FRA [53,56], given the potential impact of variations in SC SOC on the outcomes of impedance measurements. Instead, this work ensured voltage stability within a 1% tolerance by implementing a one-hour charge redistribution period prior to testing.

Correcting for the effect of parasitic impedances in the measurement circuit proved critical for eliminating high-frequency inductive artifacts. Notably, the equivalent circuit inductance decreased from 585 nH (reported in [30] for the same setup without correction) to approximately 70 nH in this work. Thus, impedance correction is essential for data reproducibility. While inductive behavior is an unwanted parasitic effect [67,68], including it in the equivalent circuit is mandatory to accurately fit the experimental frequency response.

### 4.2. Optimization of Model Parameters

The model was parametrized in a stepwise manner using linear regression and nonlinear optimization, similar to previous studies [40]. In Step 3, the algorithm performs an exhaustive sweep of initial solutions with a 1% step size relative to the previous R-L-C values. Table 11 presents the maximum errors across the full SOC range for different tolerance levels in the initial parameters.

The MSPE decreased by 3.8% when evaluating R, L, and C combinations. However, distance and angle errors increased by 1.8–13.5% and 4–5.8%, respectively; this is attributed to the objective function formulation based on relative error. Execution time increased by a factor of 200 to 11,200 during the sweep. Consequently, we selected a 5% tolerance for RLC values to trade off fitting accuracy against computational cost. To validate the proposed parameterization method, the errors for the four EDLCs are compared with the GA-based heuristic method in [29], which are shown in Table 12.

The proposed optimization algorithm reduces distance and angle errors by 51% and 15%, respectively, compared to the method described in [29]. Although the GA-based method yields a 10% lower MSPE and is approximately six times faster, the approach proposed in this work provides a superior balance between absolute and relative errors when estimating the WE-RLC model parameters, albeit at the expense of higher computational time.

### 4.3. Model Validation

The maximum distance (4.4 mΩ) and angle (5.8°) errors yielded by the proposed model are comparable to those of the WE-RLC model (4.26 mΩ and 7.1°, respectively) [30]. Regarding the MSPE, the developed variable-parameter model (3%) falls within the range of the fixed-parameter models (b) and (c) reported in [29] (1% and 7%, respectively) under varying conditions. This confirms that the impedance curve fit across multiple SOCs achieves accuracy similar to fixed-parameter SC models optimized for a single state. Consequently, the proposed model captures EDLC parameter variations across the full charge spectrum without compromising accuracy. Compared to the model presented in [40], which reports an MSPE between 0.7% and 2.6% for capacitance and resistance across different charge and frequency levels, the proposed dynamic model yields a slightly higher MSPE (2.28–3.03%) when estimating the impedance of the SC. Nevertheless, the reference model achieves that superior precision with the cost of increased complexity and the use of commercial software, which limits its accessibility and applicability.

The proposed variable parameter model reproduces the EDLC behavior across its entire SOC spectrum. However, experimental setup limitations prevented EIS measurements between 0.01 Hz and 1 Hz for bias voltages above 10 V. Consequently, the capacitance was extrapolated for 12 V and 15 V levels, which may affect model accuracy in this range. In summary, the model shows high reliability within two operating ranges: (1) from 0.01 Hz to 1 Hz for voltages between 6.25% and 62.5% of the rated value and (2) from 1 Hz to 300 kHz for voltages between 6.25% and 93.75%.

Since the model relies on small-signal testing (EIS), it is suitable for studies that aim to regulate and stabilize DC buses in the presence of small transients or perturbations. In future work, the model will be validated or improved against larger perturbations to expand its application. Future research should also address other SC technologies and cover the full frequency spectrum across all SOCs.

### 4.4. Usability of the WE-RLC Model

The BMOD0058-E016-B02 supercapacitor has been widely used for DC-link capacitance in multiple microgrids. For example, in [69], this supercapacitor is used to support the DC-link voltage of a hybrid AC-DC microgrid, where a bidirectional 3-phase inverter interfaces both AC and DC buses. Similarly, in [48] the BMOD0058-E016-B02 supercapacitor is used as DC-link between a DC microgrid and a diesel generator, which requires a bidirectional converter and a rectifier as power electronic interfaces. On the other hand, in [49,70], the BMOD0058-E016-B02 supercapacitor is used as power storage for DC microgrids. In those cases, the supercapacitors are interfaced with a bidirectional boost converter.

In the previous energy analyses, the power electronic simulations represent the BMOD0058-E016-B02 supercapacitor using the classical R-C model previously reported in Figure 1a. Some examples of power electronic simulations based on the R-C model are reported in [48,71]. Similarly, the work reported in [70] also considers the R-C model, adding a parallel resistance to simulate the self-discharge phenomena, but the calculation of such parallel resistor is not discussed. In general, the R-C model is commonly selected to perform power electronics simulations of supercapacitors due to the circuital simplicity and parameters availability, since the manufacturers usually provide both the typical capacitance and ESR values.

The main problem of performing power electronic simulations using the classical R-C model is the lack of high-frequency representation of the supercapacitor. That high-frequency response is important for testing high-frequency controllers like the ones reported in [48,49,70], which are sensible to high-frequency noise. In particular, the high-frequency switching ripple produced by DC/DC converters and inverters could be amplified by the high impedance of the supercapacitor at frequencies higher than 10 kHz (observed in Figure 13). Therefore, the operation of a switching converter interfacing a supercapacitor could be unstable due to the amplification of the high-frequency ripple produced by the real behavior of the supercapacitor. However, the power electronics simulation of such system, representing the supercapacitor with the R-C model, could predict a stable operation due to the limited representation at high-frequencies. Instead, the real behavior can be anticipated by performing the power electronics simulation with the parameterized WE-RLC model proposed in this paper.

**Figure 14:**
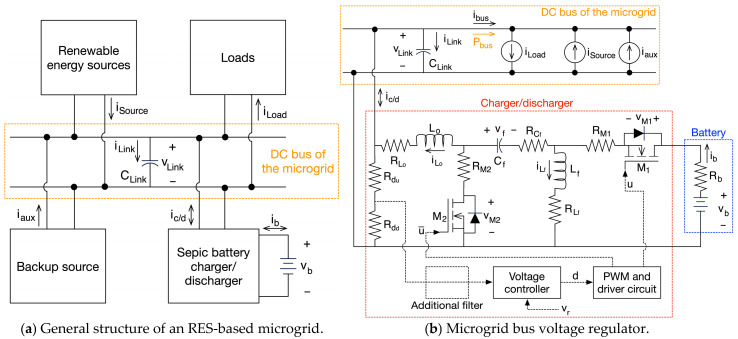
Microgrid based on renewable energy sources for model testing.

Then, the usability of the 5-parameter WE-RLC model is illustrated using a microgrid based on renewable energy sources (RESs). Figure 14a shows the general structure of the RES-based microgrid considered in this example, where the microgrid bus is formed by a supercapacitor C_Link_. The RESs provide power to the microgrid bus, and the loads consume power from that common bus. The bus voltage is regulated by a switching converter interfacing a battery; thus, the microgrid bus voltage regulator is formed by a switching converter, a control system, the supercapacitor C_Link_ and a battery. That switching converter, named charger/discharger, provides energy to the DC bus (discharging the battery) when the RES provide less power than the load consumption. Similarly, the charger/discharger stores energy extracted from the DC bus (charging the battery) when the RES provide more power than the load consumption. Finally, a backup source provides energy when the battery charge is low or when the load consumption cannot be supported with the combined power of the RES and the charger/discharger.

The detailed circuital model of the microgrid bus voltage regulator is reported in Figure 14b, where the loads are represented by a current source (i_Load_) consuming power from the DC bus, and the RES and backup source are represented by current sources (i_Source_ and i_aux_) providing power to the DC bus. Therefore, the effective current requested to the bus voltage regulator, formed by the DC-link supercapacitor (C_Link_) and charger/discharger, is i_bus_ = i_Load_ − (i_Source_ + i_aux_). This example considers a low-voltage DC bus; hence, the charger/discharger is designed with a Sepic converter. A detailed description of a Sepic-based charger/discharger is reported in [72].

The circuital simulation of the microgrid bus voltage regulator in Figure 14b is designed from realistic representations of the elements, which are selected to supply power transients up to 200 W. The considered microgrid has a DC bus voltage equal to 12 V, and due to the high availability, a 12 V battery is also selected. The inductors L_o_ and L_f_ are expected to process 25 A, then the commercial inductors PQ2614BLA-100K [73] are selected, which support up to 30 A (20% overcurrent), and have inductances equal to 10 μH and parasitic resistances of 1.29 mΩ. In stable conditions, the voltage of the intermediate capacitor C_f_ is similar to the battery voltage; hence, the commercial capacitor RKS1E180MCNAFGGS [74] is selected to support up to 25 V, thus enabling a 100% overvoltage without damage. This is an 18 μF capacitor with a series resistance 15 mΩ. The MOSFETs M_1_ and M_2_ support the sum of the battery and C_f_ voltages, i.e., 24 V; then, the commercial reference NP60N04MUK-S18-AY [75] is selected. Those MOSFETs support up to 40 V (66% overvoltage) and introduce series resistances of 3.6 mΩ and diode forward voltages of 0.9 V. The battery is designed to provide 100 times the energy stored in the supercapacitor, thus supporting a large amount of power transients. Then, the commercial battery BP10-12 [76] is selected, which has a 10 Ah capacity at v_b_ = 12 V, and introduces an ESR of 17 mΩ. Finally, two resistances YR1B20KCC [77] R_du_ = R_dd_ = 20 kΩ form a voltage divider used to measure the bus voltage. Table 13 summaries the previous commercial characteristics and the symbols used in Figure 14b.

The voltage controller in Figure 14b is designed using the small-signal model reported in [72] and the SISO tool of MATLAB [78], selecting the PI control structure described in similar applications [48,49,70]. The voltage controller includes a subtractor to calculate the error between the DC bus voltage v_Link_ and the desired value v_r_ = 12 V. Then, the PI controller processes that error to produce the duty cycle (d) for the PWM and driver circuit acting on the MOSFETs; the PWM is configured to set a 100 kHz switching frequency to the MOSFETs.

**Figure 15:**
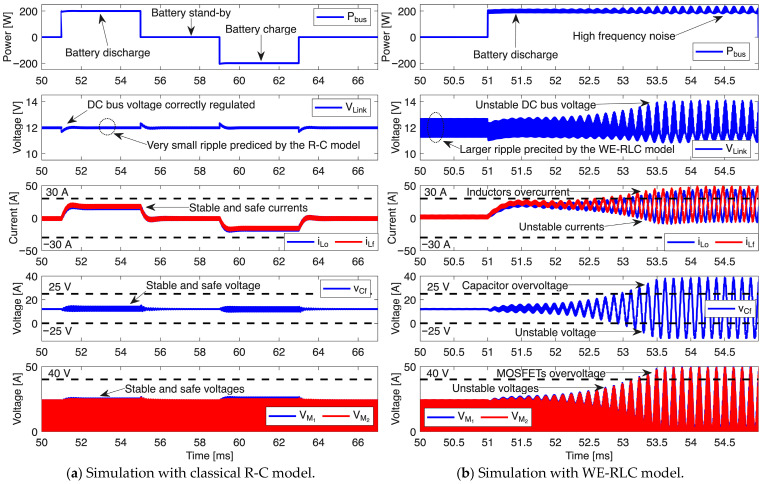
Circuital simulation of the microgrid considering R-C and WE-RLC models.

The first controller was designed to impose a settling time equal to 1 ms, resulting in G_c1_ = 0.077·(s + 9091)/s. Figure 15a reports the results of the classical simulation strategy, where the power electronics interface is tested using the classical R-C model to represent the BMOD0058-E016-B02 supercapacitor (C = 58.4 F and R = 18.5 mΩ). This first circuital simulation reports a satisfactory performance of the controller G_c1_ under three operation conditions: charger/discharger providing power to the bus (P_bus_ > 0), i.e., battery discharge; charger/discharger in stand-by mode (P_bus_ = 0); and charger/discharger storing power from the bus (P_bus_ < 0), i.e., battery charge. Figure 15a shows a correct regulation of the bus voltage (v_Link_) in all the operation conditions, achieving the desired settling time of 1 ms. In addition, this circuital simulation predicts a very small voltage ripple in the DC bus (88.5 mV) due to the small impedance of the R-C model at high frequencies. Similarly, the inductor currents (i_Lo_, i_Lf_) of the charger/discharger are stable, and both currents are below the maximum rating currents (30 A) of the PQ2614BLA-100K inductors, thus ensuring safe operation. The simulation also reports the expected stable behavior of the intermediate capacitor voltage (v_Cf_), also fulfilling the maximum voltage rating (25 V) of the RKS1E180MCNAFGGS capacitor. Finally, the bottom traces of Figure 15a show the drain–source voltages in the MOSFETs V_M1_ and V_M2_, which are both stable and under the maximum rating (40 V) of the NP60N04MUK-S18-AY MOSFETs. In conclusion, the classical simulation strategy, in which the power electronics interface is tested using the R-C model to represent the supercapacitor, predicts a stable and safe operation of the microgrid bus voltage regulator with the G_c1_ controller.

However, Figure 13 shows that the BMOD0058-E016-B02 supercapacitor has a significant increment in the impedance at high frequency; thus, the voltage ripple at the DC bus is significantly higher than the R-C model prediction, and it could be amplified by the PI controller. Therefore, the microgrid bus voltage regulator, with the same G_c1_ controller, is simulated again but representing the BMOD0058-E016-B02 supercapacitor with the proposed WE-RLC model. Figure 15b reports the results of this new simulation, where a much larger ripple at the bus voltage (15 times larger—1.35 V) is generated by the higher impedance of the supercapacitor at 100 kHz, which is reproduced by the WE-RLC model. Moreover, after the first power transient in the bus (requesting 200 W), such a large voltage ripple is amplified by the voltage controller, which makes unstable the microgrid bus voltage regulator, changing the charge condition. Similarly, the inductor currents (i_Lo_, i_Lf_) become instable, reaching up to 49.3 A, which is 97.2% higher than the expected steady-state value, and 64.3% higher than the maximum rating of the PQ2614BLA-100K inductors, thus predicting a dangerous operation condition for the inductors. The voltage of the intermediate capacitor (v_Cf_) also becomes unstable, reaching 38.7 V, which is 222.5% higher than the expected steady-state value and 54.8% higher than the maximum rating of the RKS1E180MCNAFGGS capacitor. Therefore, the intermediate capacitor also operates at a dangerous voltage condition. Finaly, the MOSFETs voltages are also unstable, reaching 50 V, which is 108.3% higher than the steady-state value, and 25% higher than the maximum voltage rating of the NP60N04MUK-S18-AY MOSFETs, hence operating in a dangerous condition. In conclusion, the circuital simulation of the power interface, including the frequency-accurate WE-RLC model, predicts an unstable operation in which the inductors, MOSFETs and intermediate capacitor operate at voltages or currents higher than the maximum rating values. Thus, this frequency-accurate simulation predicts dangerous operation conditions for the charger/discharger, which could lead to element (or complete device) destruction. This puts into evidence the importance of the WE-RLC model with voltage-dependent parameters for an accurate power electronics simulation involving supercapacitors.

**Figure 16:**
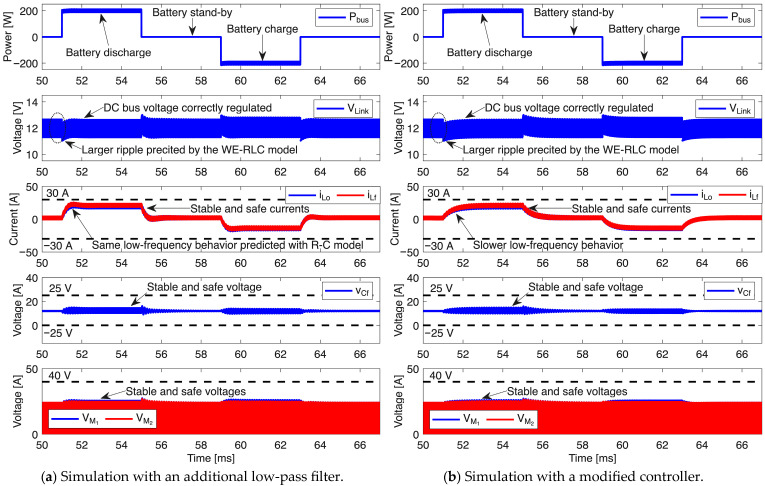
Circuital simulation of the microgrid considering solutions for high-frequency instability.

Considering the instability predicted by the circuital simulation with the proposed WE-RLC model, two solutions are available: (i) include an additional low-pass filter to attenuate the voltage ripple in the DC bus, thus requiring additional hardware, or (ii) modify the controller by reducing the gain, which introduces a low-pass filter effect at the expense of slower dynamic responses. The first solution considers the same G_c1_ controller previously tested, but it requires a low-pass filter between the voltage sensing circuit and the voltage controller. In this case, the cut-off frequency (f_c_ = 10 kHz) of the low-pass filter is set one decade below the switching frequency, which enables the attenuation of the voltage ripple in the DC bus voltage. Figure 16a shows the results of this filter-based solution, where the G_c1_ controller imposes the expected stable behavior in all the currents and voltages. Comparing the bus voltage of this simulation with the results of Figure 15a, which considers the classical R-C model, the only difference is the higher voltage ripple produced by the impedance of the supercapacitor at high frequencies, thus also imposing the designed 1 ms settling time. Similarly, the behavior of the inductor currents, intermediate capacitor and MOSFET voltages are the expected ones, where those elements operate inside the safe limits.

The second solution considers reducing the controller gain, resulting in the modified voltage controller G_c2_ = 0.032·(s + 4762)/s that imposes a longer settling time, equal to 1.5 ms. Then, the circuital simulation of the microgrid bus voltage regulator is performed again with the voltage-dependent WE-RLC model, and without any additional low-pass filter. Figure 16b shows the results of this new simulation, where the bus voltage is also stable, but the dynamic behavior is slow in comparison with the simulation of the G_c1_ controller; however, this solution does not require the additional filter; thus, less hardware is needed. The slow-dynamic behavior is also observed in the inductors’ currents, but the stable performance ensures a safe operation. This stability is also present in the intermediate capacitor and MOSFETs, where the voltages are stable and under the safe limits.

The following conclusions were obtained from the previous simulations:

- The classical simulation strategy, in which the power electronics interface is tested using the classical R-C model, is useful to define the main dynamic behavior of the system. However, the R-C model does not reproduce the impact of the high-frequency switching ripple on the controller stability.
- The proposed WE-RLC model is useful to anticipate stability problems caused by high-frequency ripples in power electronic interfaces for supercapacitors. Moreover, this WE-RLC model is useful to test practical strategies designed to mitigate such stability problems.
- Under low frequency, the WE-RLC model has a very similar behavior in comparison with the classical R-C model. Therefore, the WE-RLC model can be used to test energy management strategies designed with the classical R-C approach. However, the WE-RLC model can anticipate stability problems and high-frequency ripples that will be present at the power electronic interfaces, which is not possible with the classical R-C model.
- The parameter adaptation of the WE-RLC model is needed to test energy management systems because the supercapacitor voltage changes with the stored energy; hence, the five parameters must be adjusted following the data reported in Figure 12 and Table 9.

## 5. Conclusions

The stepwise parameterization method, based on nonlinear optimization, achieves a reasonable fit with a relative mean squared error below 3% across the entire operating range. Additionally, characterizing and removing parasitic components enabled the identification that the inductance value was affected by the distortion introduced by the experimental setup. These results address a noted limitation in the literature regarding simple, load-dependent frequency analysis models. The proposed polynomial equations predict resistive, inductive, capacitive, and diffusion (Warburg) behaviors with comparable or improved robustness relative to traditional SC models, though further validation would be beneficial.

This study offers a preliminary contribution to the optimization of energy management systems and the design of real-time controllers. The proposed variable-parameter model demonstrates reasonable predictive ability for SC behavior across various operating scenarios, which could facilitate their integration into electrical power systems; however, its general applicability requires further validation.

That is how this paper attempts to extend beyond the observation of voltage dependency by proposing an integrated methodology that combines a simplified mathematical model, a parasitic correction technique, and an optimization algorithm. The aim is to offer a potentially useful tool for the renewable energy industry. However, its accuracy and practical applicability depend on the specific operating conditions and require further experimental validation.

Future work includes exploring a model that incorporates aging and temperature variables, as well as developing a hybrid SC model that combines the empirical circuit model based on the EIS with a physico-electrochemical model, the feasibility of which remains to be determined.

## Figures and Tables

**Table 3:** Test equipment specifications.

Element	Specification	Value	Source
Supercapacitor	Rated voltage	16 V	[47]
	Rated capacitance	58 F	
	Max. ESR (initial)	22 mΩ	
	Max. stored energy	2.1 Wh	
Venable FRA 6320	Measurement accuracy	±0.03 dB + 0.1 dB/MHz ±0.4 deg + 1 deg/MHz	[51]
	Measurement bandwidth	20 MHz	
	Input range	10 mV–500 V_pk_ Full scale in 11 ranges	
	AC output amplitude	1 mV–10 V	
	DC output bias	±10 V	
Venable VLA 1500	Signal bandwidth	DC–250 kHz	[52]
	Max. input voltage	±10 V	
	Max. output voltage	17 V_RMS_ at 16 A	
	Gain	0–20 V/V	
Current sensing resistor	Resistance	102.5 Ω	Measured at 20 °C

**Table 4:** Selected test parameters.

Parameter	Value	References
Frequency range	F1: 10 mHz–1 Hz F2: 1 Hz–300 kHz	[55,56]
AC voltage amplitude	5 mV_RMS_ (F1) 10 mV_RMS_ (F2)	[53,57]
DC voltage	$[6.25–93.75\%]\;U_{N}$	[53]
Number of points	15 pts/decade	[57,58]
Integration time	2 cycles (F1) 2 s (F2)	[56]
AC stabilization time	1 cycle	[57,59]
Temperature	20 ± 2 °C	[53]

**Table 5:** Linear regression parameters to obtain initial R-L-C values.

Frequency Range (Hz)	Method	Fitted Equation
0.01–0.1	Linear regression	$Z_{SC,exp}=1/\omega C$
0.2–10k	Mean value	$Z_{SC,exp}=R$
20k–300k	Linear regression	$Z_{SC,exp}=\omega L$

**Table 6:** Fmincon parameters limits.

Variable	Lower Limit	Upper Limit
R (mΩ)	6	25
L (nH)	50	200
C (F)	35	75

**Table 7:** Lsqnonlin parameters.

Variable	Parameter	Value
$A_{W,C}$ (Ωs^−1/2^)	Lower limit	0
	Upper limit	20
	Initial value	5
$A_{W,L}$ (Ωs^−1/2^)	Lower limit	0
	Upper limit	100,000
	Initial value	200

**Table 8:** Maximum fitting errors of the WE-RLC model for SCs 1–4.

$\mathrm{SC}\;\mathrm{Voltage}\;(V,\;U_{N}$ (%))	$e_{d}$ (mΩ)	$e_{\theta }$ (°)	MSPE (%)
1 V, 6.25%	4.392	4.438	1.29
4 V, 25%	4.356	4.577	1.37
8 V, 50%	5.027	5.277	1.77
12 V, 75%	4.819	5.191	1.18
15 V, 93.75%	4.617	5.267	1.21

**Table 9:** Coefficients $a_{n}$ of the polynomial fittings of the dynamic model parameters for SC 1.

Parameter	n				
	a_4_	a_3_	a_2_	a_1_	a_0_
C (F)				1.586 · 10^0^	4.084 · 10^1^
R (mΩ)		6.146 · 10^4^	−1.086 · 10^−2^	−4.269 · 10^−2^	1.234 · 10^1^
L (nH)	−2.752 · 10^−3^	7.560 · 10^−2^	−6.103 · 10^−1^	1.833 · 10^0^	6.679 · 10^1^
A_W,C_ (Ωs^−1/2^)		6.905 · 10^−3^	−1.271 · 10^−1^	6.537 · 10^−1^	3.017 · 10^−1^
A_W,L_ (Ωs^−1/2^)		3.387 · 10^0^	−1.018 · 10^1^	−3.744 · 10^1^	1.458 · 10^3^

**Table 10:** Error values of the dynamic supercapacitor model for SC 1 at different bias voltages.

$\mathrm{SC}\;\mathrm{Voltage}\;(V,\;U_{N}$ (%))	$e_{d}$ (mΩ)	$e_{\theta }$ (°)	MSPE (%)
1 V, 6.25%	4.220	4.359	2.34
4 V, 25%	4.401	4.517	2.55
8 V, 50%	4.355	4.990	3.03
12 V, 75%	2.805	5.752	2.28
15 V, 93.75%	3.810	5.487	2.94

**Table 11:** Sensitivity analysis of the WE-RLC model error to the initial solution.

Tolerance Range	$e_{d}$ (mΩ)	$e_{\theta }$ (°)	MSPE (%)	Elapsed Time (s)
±0%	4.938	5.072	1.82	0.142
±5%	5.027	5.277	1.77	28.2
±10%	5.602	5.366	1.75	194
±15%	5.602	5.366	1.75	672
±20%	5.602	5.366	1.75	1590

**Table 12:** Fitting errors of different parameterization methods.

Method	$e_{d}$ (mΩ)	$e_{\theta }$	MSPE (%)	Elapsed Time (s)
[29]	7.604	6.052	1.59	3.91
This work	5.027	5.277	1.77	25.11

**Table 13:** Commercial parameters for the circuital simulations.

Element	Parameter	Symbol	Value
DC bus	Voltage	v_Link_	12 V
Battery (BP10-12)	Voltage	v_b_	12 V 17 mΩ
	ESR	R_b_	
Inductors (PQ2614BLA-100K)	Inductance	L_o_, L_f_	10 μH 1.29 mΩ 30 A
	ESR	R_Lo_, R_Lf_	
	Maximum current	-	
Intermediate capacitor (RKS1E180MCNAFGGS)	Capacitance ESR Maximum voltage	C_f_ R_Cf_ _-_	18 μF 15 mΩ 25 V
MOSFETs (NP60N04MUK-S18-AY)	ON resistance Diode forward voltages Maximum voltage	R_M1_, R_M2_ (inside the MOSFET model) -	3.6 mΩ 0.9 V 40 V
DC bus voltage measurement (YR1B20KCC)	Resistive divider	R_du_, R_dd_	20 kΩ

**Figure A1:**
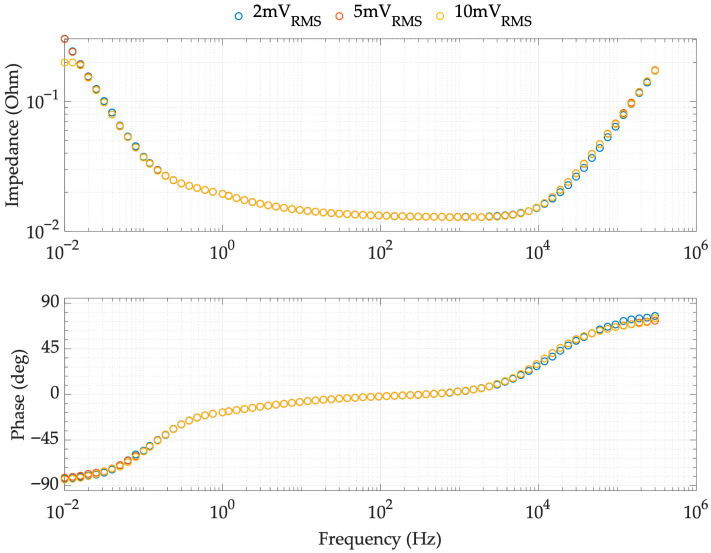
Frequency response of SC 1 at 8 V and different AC perturbation amplitudes.

**Figure A2:**
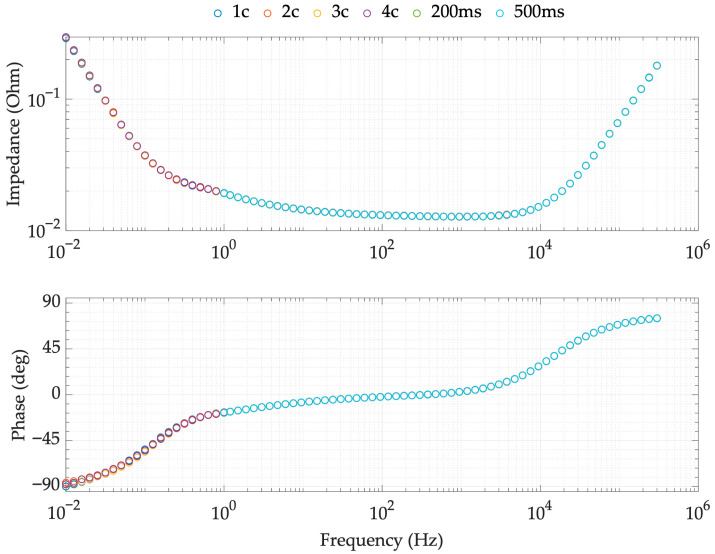
Frequency response of SC 1 at 8 V and different integration times.

**Figure A3:**
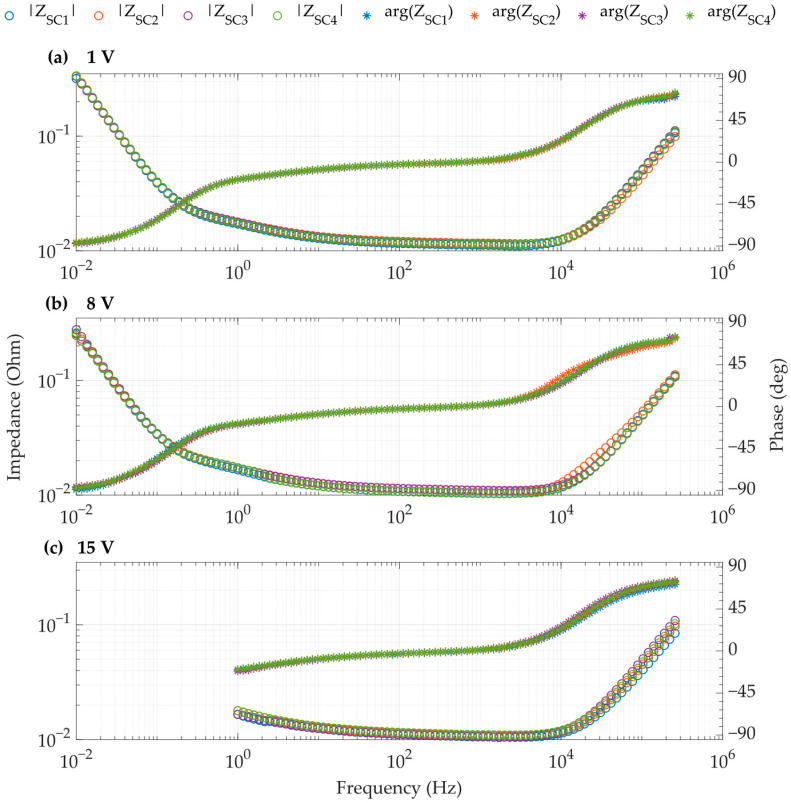
Frequency response of SCs 1–4 at bias voltages of (**a**) 1 V; (**b**) 8 V; and (**c**) 15 V.

## Data Availability

The datasets presented in this article are not readily available because the data are part of an ongoing study.

## Abbreviations

The following abbreviations are used in this manuscript:

AC	Alternating current
CART	Classification and regression tree
CTRR	Conventional trust region reflection
CV	Cyclic voltammetry
DC	Direct current
EDLC	Electrochemical double-layer capacitor
EIS	Electrochemical impedance spectroscopy
ESR	Equivalent series resistor
FRA	Frequency response analyzer
GA	Genetic algorithm
GCD	Galvanostatic charge–discharge
GRG	Generalized reduced gradient
LFP	Lithium iron phosphate
LSO	Least squares optimization
LTO	Lithium titanate oxide
ML	Machine learning
NLO	Non-linear optimization
NMC	Nickel manganese cobalt
PSO	Particle swarm optimization
RF	Random forest
RLS	Recursive least squares
RSS	Residual sum of squares
SC	Supercapacitor
SOC	State of charge
SNR	Signal to noise ratio
SSID	Subspace System Identification
VLA	Voltage linear amplifier
WE-RLC	Warburg-Enhanced RLC model

## Appendix A

The SC response during the EIS must be linear and stable (independent of time and perturbation amplitude). To verify linearity, the SC 1 was tested at 8 V using an integration time of one cycle and various perturbation amplitudes. Figure A1 shows the results.

In the frequency range of 1 Hz to 10 kHz, SC 1 exhibits similar impedance at amplitudes of 2 mV, 5 mV, and 10 mV. However, below 1 Hz, the response at 10 mV shows a maximum relative difference of 34% compared to the impedance at 2 mV (in contrast to the 1.6% difference observed at 5 mV). This demonstrates that, at low frequencies, an amplitude of 10 mV induces a nonlinear response of the SC during EIS measurements, whereas the responses at 2 mV and 5 mV remain similar. In contrast, at frequencies above 10 kHz, the behavior at 5 mV and 10 mV is similar: the maximum difference from the data at 10 mV is 2% (compared to 7.4% for the 2 mV amplitude). This discrepancy is attributed to the noise increment at high frequencies; consequently, signals of higher amplitude (5 and 10 mV) exhibit enhanced SNR, facilitating more precise reproduction and analysis.

To verify the stability of the SC frequency response, the SC 1 was tested at 8 V using a perturbation amplitude of 5 mV and integration times of 1, 2, 3, and 4 cycles, as well as 200 ms and 500 ms. Figure A2 shows the results.

The integration times of 1 cycle, 2 cycles, 200 ms, and 500 ms were evaluated in the frequency range of 1 to 300 kHz. Within this interval, the maximum variance from the mean value was 0.6% for magnitude and 0.19° for phase. Consequently, impedance remains independent of the integration time, demonstrating the high repeatability of the results.

The integration times of 1, 2, 3, and 4 cycles were evaluated in the frequency range of 10 mHz to 1 Hz. The maximum variance from the mean value was 1.3% for magnitude and 2° for phase. This increased dispersion of the results at low frequencies occurs because the duration of the test multiplies as the number of integration cycles increases. Consequently, longer durations amplify the drift of the bias voltage and other unwanted effects during the execution of EIS. These effects may result from a lack of calibration or from the inherent uncertainty of the values configured in the test equipment. Therefore, given the repeatability of the tests, we recommend using an integration time of one or two cycles at low frequencies.

## Appendix B

The frequency response results for SC 1 are depicted in Figure 9 for bias voltages of 1 V, 4 V, 8 V, 12 V, and 15 V. Figure A3 reports the Bode plots obtained for the four tested SCs at 1 V, 8 V, and 15 V.

The four studied SCs exhibited similar behavior at frequencies below 1 kHz. However, at higher frequencies, impedance showed higher dispersion. This is attributed to parasitic inductive behavior, which depends on the EDLC construction and ion–pore interaction. Furthermore, dispersion increased at high SOC levels, suggesting an alteration in the ion–pore interaction and the dominance of charge redistribution mechanisms [79].

## References

[1] IEA (2025). Renewables 2025. https://www.iea.org/reports/renewables-2025.

[2] Ejuh Che E., Roland Abeng K., Iweh C.D., Tsekouras G.J., Fopah-Lele A. (2025). The Impact of Integrating Variable Renewable Energy Sources into Grid-Connected Power Systems: Challenges, Mitigation Strategies, and Prospects. Energies.

[3] Singh S., Singh S. (2024). Advancements and Challenges in Integrating Renewable Energy Sources Into Distribution Grid Systems: A Comprehensive Review. J. Energy Resour. Technol. Trans. ASME.

[4] Deguenon L., Yamegueu D., Moussa kadri S., Gomna A. (2023). Overcoming the Challenges of Integrating Variable Renewable Energy to the Grid: A Comprehensive Review of Electrochemical Battery Storage Systems. J. Power Sources.

[5] Hossain Lipu M.S., Rahman M.S.A., Islam Z.U., Rahman T., Rahman M.S., Meraj S.T., Hossain M.Y., Mansor M. (2025). Review of Energy Storage Integration in Off-Grid and Grid-Connected Hybrid Renewable Energy Systems: Structures, Optimizations, Challenges and Opportunities. J. Energy Storage.

[6] Mastoi M.S., Wang D., Zhou X., He X., Hassan M., Ali A., Rehman A. (2025). Study of Energy Storage Technology Approaches for Mitigating Wind Power Fluctuations to Enhance Smart Grid Resilience. Renew. Sustain. Energy Rev..

[7] Taghizad-Tavana K., Esmaeel Nezhad A., Hagh M.T., Canani A., Safari A. (2025). Hybrid Renewable Energy Systems for Off-Grid Electrification: A Comprehensive Review of Storage Technologies, Metaheuristic Optimization Approaches and Key Challenges. Eng.

[8] Aguado Molina R., Cartelle Barros J.J., de la Cruz López M.d.P., Lara Coira M., del Caño Gochi A. (2025). A Comparative Sustainability Assessment of Several Grid Energy Storage Technologies. Appl. Energy.

[9] Samy M.M., Güven A.F. (2025). Optimal Dimensioning of Grid-Connected PV/Wind Hybrid Renewable Energy Systems with Battery and Supercapacitor Storage a Statistical Validation of Meta-Heuristic Algorithm Performance. Sci. Rep..

[10] Shuvo J.I., Badoruzzaman M., Anik S.T.I., Ahmad S., Ahmed T., Karimi M. (2025). Hybrid Energy Storage Power Management System Harnessing Battery-Supercapacitor Synergy for Grid-Isolated DC Microgrid. J. Energy Storage.

[11] Aslam H., Umar A., Khan M.U., Trivedi T., Ezhilarasan G., Bhanot D., Abbas N., Fawy K.F. (2025). Recent Trends in Supercapacitor Technology; Basics, History, Fabrications, Classifications and Their Application in Energy Storage Materials. Rev. Inorg. Chem..

[12] Salaheldeen M., Eskander T.N.A., Fathalla M., Zhukova V., Blanco J.M., Gonzalez J., Zhukov A., Abu-Dief A.M. (2025). Empowering the Future: Cutting-Edge Developments in Supercapacitor Technology for Enhanced Energy Storage. Batteries.

[13] Volfkovich Y.M. (2024). Electric Double Layer Capacitors: A Review. Russ. J. Electrochem..

[14] Wei Y.M., Kumar K.D., Zhang L., Li J.F. (2025). Pseudocapacitive Materials for Energy Storage: Properties, Mechanisms, and Applications in Supercapacitors and Batteries. Front. Chem..

[15] Parvin N., Merum D., Kang M., Joo S.W., Jung J.H., Mandal T.K. (2025). Recent Advances in Hybrid Supercapacitors: A Review of High Performance Materials and Scalable Fabrication Techniques. J. Mater. Chem. A Mater..

[16] Libich J., Máca J., Vondrák J., Čech O., Sedlaříková M. (2018). Supercapacitors: Properties and Applications. J. Energy Storage.

[17] Kumar N., Kim S.B., Lee S.Y., Park S.J. (2022). Recent Advanced Supercapacitor: A Review of Storage Mechanisms, Electrode Materials, Modification, and Perspectives. Nanomaterials.

[18] Zhao J., Burke A.F. (2021). Review on Supercapacitors: Technologies and Performance Evaluation. J. Energy Chem..

[19] Vega-Muratalla V.O., Serrano-Arévalo T.I., Ochoa-Barragán R., Lira-Barragán L.F., Ramírez-Márquez C., El-Halwagi M.M., Ponce-Ortega J.M. (2026). Recent Advances and Engineering Challenges of Lithium Batteries for Grid-Level Energy Storage: A Review. Ind. Eng. Chem. Res..

[20] Yuan F., Zhou J., Yamauchi Y., Wu Y., Wan C. (2026). Supercapacitor Dynamics: Mechanisms, Architectures, and Advanced in-Situ Characterizations for next-Generation Energy Storage. Coord. Chem. Rev..

[21] Couto L.D., Charkhgard M., Karaman B., Job N., Kinnaert M. (2023). Lithium-Ion Battery Design Optimization Based on a Dimensionless Reduced-Order Electrochemical Model. Energy.

[22] Nemeth T., Schröer P., Kuipers M., Sauer D.U. (2020). Lithium Titanate Oxide Battery Cells for High-Power Automotive Applications—Electro-Thermal Properties, Aging Behavior and Cost Considerations. J. Energy Storage.

[23] Zhang Y., Li X., Li Z., Yang F. (2024). Evaluation of Electrochemical Performance of Supercapacitors from Equivalent Circuits through Cyclic Voltammetry and Galvanostatic Charge/Discharge. J. Energy Storage.

[24] Bograchev D.A., Volfkovich Y.M., Martemianov S. (2023). Diagnostics of Supercapacitors Using Cyclic Voltammetry: Modeling and Experimental Applications. J. Electroanal. Chem..

[25] Yun C., Hwang S. (2020). Analysis of the Charging Current in Cyclic Voltammetry and Supercapacitor’s Galvanostatic Charging Profile Based on a Constant-Phase Element. ACS Omega.

[26] Santa-Cruz L.A., Tavares F.C., Loguercio L.F., dos Santos C.I.L., Galvão R.A., Alves O.A.L., Oliveira M.Z., Torresi R.M., Machado G. (2024). Electrochemical Impedance Spectroscopy: From Breakthroughs to Functional Utility in Supercapacitors and Batteries—A Comprehensive Assessment. Phys. Chem. Chem. Phys..

[27] Azizpour A., Bagovic N., Ploumis N., Mylonas K., Hoxha D., Kienberger F., Al-Zubaidi-R-Smith N., Gramse G. (2025). Electrochemical Analysis of Carbon-Based Supercapacitors Using Finite Element Modeling and Impedance Spectroscopy. Energies.

[28] Ma N., Yang D., Riaz S., Wang L., Wang K. (2023). Aging Mechanism and Models of Supercapacitors: A Review. Technologies.

[29] Lozito G.M., Intravaia M., Corti F., Patrizi G., Laschi M., Ciani L., Vangi D., Reatti A. (2024). Equivalent Circuit Modelling of Hybrid Supercapacitors Through Experimental Spectroscopic Measurements. IEEE Access.

[30] Rus-Casas C., Ramos-Paja C.A., Serna-Garcés S.I., Gilabert-Torres C., Aguilar-Peña J.D. (2025). A Circuital Equivalent for Supercapacitors Accurate Simulation in Power Electronics Systems. Batteries.

[31] Randles J.E.B. (1947). Kinetics of Rapid Electrode Reactions. Discuss. Faraday Soc..

[32] Nguyen T.Q., Breitkopf C. (2018). Determination of Diffusion Coefficients Using Impedance Spectroscopy Data. J. Electrochem. Soc..

[33] Naseri F., Karimi S., Farjah E., Schaltz E. (2022). Supercapacitor Management System: A Comprehensive Review of Modeling, Estimation, Balancing, and Protection Techniques. Renew. Sustain. Energy Rev..

[34] Sedlakova V., Sikula J., Majzner J., Sedlak P., Kuparowitz T., Buergler B., Vasina P. (2015). Supercapacitor Equivalent Electrical Circuit Model Based on Charges Redistribution by Diffusion. J. Power Sources.

[35] Zucca M., Hassanzadeh M., Conti O., Pogliano U. (2023). Accurate Parameters Identification of a Supercapacitor Three-Branch Model. IEEE Access.

[36] Slaifstein D., Ibanez F.M., Siwek K. (2023). Supercapacitor Modeling: A System Identification Approach. IEEE Trans. Energy Convers..

[37] Boby R.M., Rahman S.H., Radhakrishnan R. (2025). A Variable Capacitance Based Modelling and Parameter Determination for Supercapacitor with Experimental Validation. 2025 International Conference on Power Electronics Converters for Transportation and Energy Applications, PECTEA 2025.

[38] Yasin A., Dhaouadi R., Mukhopadhyay S. (2024). A Novel Supercapacitor Model Parameters Identification Method Using Metaheuristic Gradient-Based Optimization Algorithms. Energies.

[39] Maity S., Saha M., Saha P., Khanra M. (2024). Fractional Calculus-Based Modeling and State-of-Charge Estimation of Supercapacitor. J. Energy Storage.

[40] Navarro G., Nájera J., Torres J., Blanco M., Santos M., Lafoz M. (2020). Development and Experimental Validation of a Supercapacitor Frequency Domain Model for Industrial Energy Applications Considering Dynamic Behaviour at High Frequencies. Energies.

[41] Hardianto Y.P., Shah S.S., Shuaibu A.D., Mohamed M., Sarker S., Alzahrani A.S., Aziz M.A. (2025). Modeling Supercapacitors with the Simplified Randles Circuit: Analyzing Electrochemical Behavior through Cyclic Voltammetry and Galvanostatic Charge-Discharge. Electrochim. Acta.

[42] Hardianto Y.P., Mirghni A.A., Shah S.S., Sarker S., Aziz M.A. (2025). Developing an Innovative Theoretical Model for Analyzing Cyclic Voltammetry in Hybrid Supercapacitors. Electrochim. Acta.

[43] Shelake A.R., Karade V.C., Selvaraj M., Assiri M.A., More D.D., Patil A.D., Mali M.G., Sutar S.S., Amate R.U., Jeon C.-W. (2026). Machine Learning-Guided Optimization, Predictive Modeling, and Experimental Validation of MXene-Based Supercapacitors. J. Power Sources.

[44] Morandi A., Lampasi A., Cocchi A., Gherdovich F., Melaccio U., Ribani P.L., Rossi C., Soavi F. (2021). Characterization and Model Parameters of Large Commercial Supercapacitor Cells. IEEE Access.

[45] Rahman S.H., Jagadanand J.G., Saju P.V., Shreelakshmi M.P. (2025). Real Time Terminal Voltage Prediction and State-of-Charge Estimation for Supercapacitors Using Fractional Order Model. IEEE Trans. Power Electron..

[46] Tshiani C.T., Umenne P. (2022). The Characterization of the Electric Double-Layer Capacitor (EDLC) Using Python/MATLAB/Simulink (PMS)-Hybrid Model. Energies.

[47] Datasheet of Maxwell BMOD0058E016B02- 16V Small Cell Module 2014, 1015371.6. https://maxwell.com/wp-content/uploads/2021/08/3003212.2_Datasheet_BMOD0058-E016-C02.pdf.

[48] Pannala S., Padhy N.P., Agarwal P. (2020). Effective Power Management Scheme for PV-Battery-DG Integrated Standalone DC Microgrid. IET Electr. Power Appl..

[49] Behera P.K., Pattnaik M. (2024). Supervisory Power Management Scheme of a Laboratory Scale Wind-PV Based LVDC Microgrid Integrated With Hybrid Energy Storage System. IEEE Trans. Ind. Appl..

[50] Karpana S., Batzelis E., Maiti S., Chakraborty C. (2022). PV-Supercapacitor Tri-Port Converter for Frequency Response Services. 10th IEEE International Conference on Power Electronics, Drives and Energy Systems, PEDES 2022.

[51] (2025). Venable Instruments 63XX Frequency Response Analyzer. Venable Instruments 09052024. https://www.scribd.com/document/877968640/Venable-Instruments-63XX-FRA-Data-Sheet-09052024-1.

[52] (2023). Venable Instruments VLA1500/2500—Operator’s Manual. Venable Instruments 96-8006861_1-31. https://www.venableinstruments.com/hubfs/Manuals/Venable%20Software%20Manual%206_7-1.pdf.

[53] (2022). Fixed Electric Double-Layer Capacitors for Use in Electric and Electronic Equipment—Part 1: Generic Specification.

[54] (2025). Fixed Electric Double-Layer Capacitors for Use in Electronic Equipment—Part 2: Sectional Specification—Electric Double-Layer Capacitors for Power Application.

[55] Macdonald J.R., Johnson W.B., Raistrick I.D., Franceschetti D.R., Wagner N., McKubre M.C.H., Macdonald D.D., Sayers B., Bonanos N., Steele B.C.H. (2018). Impedance Spectroscopy: Theory, Experiment, and Applications.

[56] Lazanas A.C., Prodromidis M.I. (2023). Electrochemical Impedance Spectroscopy—A Tutorial. ACS Meas. Sci. Au.

[57] Pedro Aguiar dos Santos J., Cesar Rufino F., Yutaka Ota J.I., Fernandes R.C., Vicentini R., Pagan C.J.B., Morais Da Silva L., Zanin H. (2023). Best Practices for Electrochemical Characterization of Supercapacitors. J. Energy Chem..

[58] Metrohm Electrochemical Impedance Spectroscopy (EIS). Part 2—Experimental Setup. https://www.metrohm.com/en/applications/application-notes/autolab-applikationen-anautolab/an-eis-002.html.

[59] Pollard R., Comte T. (1989). Determination of Transport Properties for Solid Electrolytes from the Impedance of Thin Layer Cells. J. Electrochem. Soc..

[60] Abd El-Latif E.I., Kebede A., Sekar K., Hameed T.A., Yahia I.S., Gao H., Sheha E., El-Latif E.I.A., Kebede M.A. (2025). Modeling the Diffusion Coefficient of Charge Carriers in Metal Ion Batteries Using the Randles-Sevcik Equation. Adv. Theory Simul..

[61] Huang J. (2018). Diffusion Impedance of Electroactive Materials, Electrolytic Solutions and Porous Electrodes: Warburg Impedance and Beyond. Electrochim. Acta.

[62] Yang H. (2020). A Comparative Study of Supercapacitor Capacitance Characterization Methods. J. Energy Storage.

[63] Lin T., Ma S., Ye Y., Zhang S. (2021). An ADMM-Based Interior-Point Method for Large-Scale Linear Programming. Optim. Methods Softw..

[64] Volfkovich Y.M., Bograchev D.A., Mikhalin A.A., Bagotsky V.S. (2013). Supercapacitor Carbon Electrodes with High Capacitance. J. Solid State Electrochem..

[65] Mohammad Naim N.N. (2015). Modelling the Ageing Behaviour of Supercapacitors Using Electrochemical Impedance Spectroscopy for Dynamic Applications. Ph.D. Dissertation.

[66] Pameté E., Köps L., Kreth F.A., Pohlmann S., Varzi A., Brousse T., Balducci A., Presser V. (2023). The Many Deaths of Supercapacitors: Degradation, Aging, and Performance Fading. Adv. Energy Mater..

[67] Quintanal N., Barreda D., Blanco C., González Z., Álvarez P., Granda M., Sevilla M., Santamaría R. (2023). An Insight into the Mechanisms of Energy Storage in a Double Layer Capacitor with ILs and a Microporous Carbon: Experimental Evidences of Ion-Swapping by Electrochemical Impedance Spectroscopy. J. Electrochem. Soc..

[68] Bisquert J., Guerrero A. (2022). Chemical Inductor. J. Am. Chem. Soc..

[69] Habib H.F., Mohamed A.A.S., El Hariri M., Mohammed O.A. (2017). Utilizing Supercapacitors for Resiliency Enhancements and Adaptive Microgrid Protection against Communication Failures. Electr. Power Syst. Res..

[70] Challouf I., Khemissi L., Gannouni F., Rehaoulia A., Sellami A., Ben Hmida F., Bouaicha M. (2025). Hybrid Supercapacitor–Battery System for PV Modules Under Partial Shading: Modeling, Simulation, and Implementation. Energies.

[71] Cabello J.M., Roboam X., Junco S., Turpin C. (2019). Direct Sizing and Characterization of Energy Storage Systems in the Energy-Power Plane. Math. Comput. Simul..

[72] Montenegro-Oviedo J.A., Ramos-Paja C.A., Orozco-Gutierrez M.L., Franco-Mejía E., Serna-Garcés S.I. (2023). Adaptive Controller for Bus Voltage Regulation on a DC Microgrid Using a Sepic/Zeta Battery Charger/Discharger. Mathematics.

[73] Bourns (2025). PQ2614BLA/BHA Series-Shielded Power Inductors. https://www.bourns.com/docs/product-datasheets/pq2614.pdf.

[74] Nichicon Corporation Conductive Polymer Aluminum Solid Electrolytic Capacitors—FPCAP Series (CAT.8100M). https://www.nichicon.com/getmedia/8453d4c3-b1c6-4d18-bcbb-a2b3657e523e/e-polymer.pdf.

[75] Renesas Electronics Corporation (2018). NP60N04MUK, NP60N04NUK; MOS Field Effect Transistor. R07DS0597EJ0200. https://www.renesas.com/en/document/dst/np60n04muk-np60n04nukmos-field-effect-transistor?srsltid=AfmBOorX6yhVM43Up1EVD5UKj9Q6tjJSOgKbmcAZz1Xb0eHpuZg9RY0B.

[76] BB Battery BP10-12-12V10.0AH. https://www.bb-bat.com/en/product/BP1012.html.

[77] TE Connectivity YR1B20KCC: NEOHM Through-Hole Resistors. https://www.te.com/en/product-3-1676913-0.html.

[78] MathWorks Control System Designer—Design Single-Input, Single-Output (SISO) Controllers—MATLAB. https://www.mathworks.com/help/control/ref/controlsystemdesigner-app.html.

[79] Haque M., Li Q., Rigato C., Rajaras A., Smith A.D., Lundgren P., Enoksson P. (2021). Identification of Self-Discharge Mechanisms of Ionic Liquid Electrolyte Based Supercapacitor under High-Temperature Operation. J. Power Sources.

